# Editorial: Exploring the interplay between microbiota, gut, and brain in inflammatory bowel disease

**DOI:** 10.3389/fneur.2026.1810252

**Published:** 2026-03-18

**Authors:** Le Liu

**Affiliations:** Department of Gastroenterology, Zhujiang Hospital, Southern Medical University, Guangzhou, Guangdong, China

**Keywords:** digital therapeutics, endocannabinoidome, inflammatory bowel disease, microbiota-gut-brain axis, neuroimmune signaling

Inflammatory bowel disease (IBD) is increasingly understood as a condition in which intestinal inflammation, barrier function, microbial ecology, and neuroimmune signaling interact to shape both disease activity and lived symptoms. Beyond diarrhea and bleeding, many patients experience abdominal pain, fatigue, sleep disturbance, and mood symptoms that can persist even when inflammation is controlled and may not fully track objective inflammatory activity (1, 2). Recent findings endorse a bidirectional microbiota-gut-brain framework in IBD. Psychological distress and central stress circuits may further exacerbate IBD symptom burden and affect physiology. Similarly, gut inflammation, dysbiosis and microbial metabolites may alter vagal and immune signaling with downstream modulations of the processing of pain, affect, and behavior (1–3). Experimental studies further highlight microbe-derived extracellular vesicles as a plausible mediator linking intestinal inflammation to brain outcomes, specifically colitis-associated cognitive impairment (4, 5). Preclinical evidence helps identify possible pathways via which the gut inflammation may manifest in measurable neurobehavioral outcomes. This Research Topic aimed to facilitate discussions that are limited to the terms “microbiome,” “gut” and “brain,” to highlight interfaces that are mechanistically and clinically actionable.

The Research Topic brings together five contributions spanning molecular perspectives, digital behavioral intervention, patient decision-making, and neurorehabilitation. These articles imply that gut-brain concepts become clinically useful only when they are linked to mechanisms, scalable interventions, and practical phenotyping that separates inflammatory from functional or neurogenic drivers of symptoms. A mechanistic anchor is provided by a review of the endocannabinoidome (eCBome) as a signaling network linking microbial cues, epithelial barrier integrity, immune tone, and neurobehavioral regulation (Wang et al.). This view is helpful as it reinterprets the endocannabinoid-related mediators as components in a lipid and receptor system that responds to diet, exercise, and microorganisms. In IBD, where the same patient may have inflammatory activity, visceral hypersensitivity, and stress-related symptom amplification, the eCBome may provide a plausible point of convergence for interventions that combined lifestyle modification and targeted pharmacology, while also sharpening hypotheses about microbial-host communication.

Clinical translation, however, requires interventions that patients can access and sustain. A randomized controlled trial evaluated a brief, fully online acceptance and commitment therapy program (iACT4IBD) for adults with IBD (Lin et al.). The trial, although not showing short-term superiority for psychological outcomes, nevertheless offers an important learning lesson. In an important point, the symptom burden and quality-of-life outcomes in IBD frequently do not align with objective inflammatory markers, therefore trials targeting gut-brain processes may require endpoints and follow-up windows capturing this dissociation (1, 2). Initially, the scalable delivery of gut-brain interventions would allow for digital implementation which is a critical factor for IBD populations with time, distance and health variability barriers. Following that, indications in physical results including Crohn's disease activity give backing to move ahead with work on optimizing/dosing, involvement support, and measuring and measuring approaches. In investigations surrounding the gut-brain axis, if a clinical outcome does not yield any positive confirmation or a combination of results yield null results it should not be interpreted as a failure of the framework. Rather, it should be considered as useful evidence toward either better stratification or more sensitive endpoints or a combination thereof. It also deserves to note that results can be improved by better integration with biological measures that can capture stress-immune coupling (6).

A complementary translation gap is addressed by an assessment of knowledge, attitudes, and decisional conflict regarding biologics among patients with IBD (Li et al.). According to their findings, when effective treatments exist, uncertainty and limited knowledge of treatment can restrict shared decision-making. From the viewpoint of microbiota-gut-brain interactions, these processes are not marginal. Decisional conflict can increase anticipatory anxiety, disrupt adherence, and heighten vigilance to symptoms, which may increase the perceived burden of disease and complicate symptom-inflammation matching. Thus, an important and modifiable target linking clinical outcomes and brain-gut pathways is the improvement of biologic literacy and decisional support, especially in situations with limited health literacy and access to structured counseling.

Two further contributions widen the focus to illustrate how brain-to-gut pathways can dominate bowel function and how neuroimmune disease can present at the extremes of age. A national survey described clinician perspectives and practices in managing bowel disorders in severe acquired brain injury during neurorehabilitation (Intiso et al.). While the study does not focus on IBD, it will highlight how neurological disruption, immobility, medications and altered autonomic control can produce significant bowel dysfunction. However, the assessment and management vary appreciably. For clinicians dealing with IBD, the issue is relevant both conceptually and practically since central and autonomic factors can drive bowel symptoms. Standardized assessment that distinguishes inflammatory activity from the functional and neurogenic contributions serves as useful care pathways. A case report and review highlights very-late-onset neuromyelitis optica spectrum disorder (Liu et al.). This report underscores that immune-mediated central nervous system disease can emerge late in life and respond to targeted immunotherapy, encouraging continued attention to neuroimmune susceptibility as IBD populations age and as systemic immune modulation becomes more common. As immunomodulatory options continue to expand, these therapies intersect with gut-brain axis biology and microbiota-targeted strategies, reinforcing the value of tracking neurobehavioral outcomes alongside inflammatory control (7). Though not specific to IBD, these studies are “boundary cases” that clarify how autonomic disruption, medication exposure and neuroimmune activity can all independently change bowel function and patient experience. These themes recur in IBD care as well. Cumulatively, the two contributions make a practical point for gut-brain work in IBD: bowel symptoms and quality-of-life impairment may be caused by central or autonomic dysfunction, drug effects, or another neuroimmune response, and diagnostic misclassification can occur if all the symptom change is attributed to intestinal inflammation (6, 8).

Placed in broader context, the Research Topic aligns with several convergent directions in the field. Over time, mechanistic models have progressed from simple descriptive association to pathways involving vagal afferents, cytokines, microbial metabolites, and neuroactive compounds that are testable. Evidence from related gut-brain conditions with neuroimmune and functional involvement can assist in the prioritization of microbial candidates and classes of mediator for later testing in IBD-specific cohorts. Research involving other neuroimmune areas provides further evidence that specific strains of gut microbes may ameliorate neuroinflammation through gut-brain signaling. For instance, *Parabacteroides goldsteinii* was shown to reduce neuroinflammation in LRRK2 mutant mice and ameliorate parkinsonism phenotypes (9). A study conducted on an irritable bowel syndrome model shows that a GABA-producing *Lactococcus lactis* strain alleviates intestinal impairment and ameliorates neurobehavioral anomalies in the model, providing evidence that microbe-derived neurotransmitters act as functional mediators of gut-brain communication (10). Microbiota-gut-brain research increasingly relies on measurement, including tools that better assess intestinal barrier status and mucosal integrity, which help link gut states to extraintestinal manifestations and patient reported outcomes (11). Meanwhile, it is noteworthy that dietary strategies and nutritional therapies are also being reframed not only as supportive care, but as biologically plausible modifiers of microbial ecology and symptom pathways (12). Ultimately, neuromodulation is moving from concept to early proof-of-principle. Pilot data on vagus nerve stimulation in Crohn's disease support the possibility that altering autonomic signaling may influence inflammatory pathways, while reinforcing the need for robust trials and careful patient selection (13). Recent reviews also highlight that neuropsychiatric comorbidity in IBD may be both a consequence and a driver of disease burden, providing further grounds for microbiota-gut-brain sensitive integrated care (14, 15).

Looking forward, three priorities could accelerate clinical impact. A central challenge is disentangling directionality and confounding, since inflammation, diet, medication exposure, and stress can all reshape the microbiome and symptoms simultaneously. First, mechanistic stratification is essential. Rather than treating “IBD with pain” or “IBD with anxiety” as uniform categories, future studies should combine microbiome and metabolome profiling with inflammatory markers, barrier measures, and neurobehavioral phenotyping to define treatable subtypes. In practice, this stratification could incorporate pathways highlighted by recent preclinical work, such as microbiota-derived extracellular vesicles and tryptophan-related metabolic signatures, as well as microbe-derived neuroactive mediators including GABA, to generate biomarker-linked, testable intervention targets (5, 10). Second, endpoints should reflect the gut-brain axis as a system. Trials should pair validated patient-reported outcomes with biomarkers of inflammation and barrier function, and when feasible include measures that capture central processing, such as cognitive tasks or neuroimaging. Third, implementation must be designed for real-world care. The assessment of digital therapeutics, structured decisional support, and integrated psychological pathways must be done while taking adherence, equity, and cultural tailoring into consideration so that microbiota-gut-brain science can be translated. More importantly, it would be translatable to usable clinical routines.

In short, our Research Topic indicates that microbiota-gut-brain interactions in IBD are not just a concept but a translational framework involving molecular signals, symptoms, treatment choices and consequences. The Research Topic, which aims to promote the next generation of biologically plausible and clinically relevant interdisciplinary research, does so by bringing together work on the microbiota-eCBome interface, scalable behavioral intervention, patient decisional conflict, and clinically meaningful brain-to-gut examples.
